# The Ant Who Cried Wolf? Short-Term Repeated Exposure to Alarm Pheromone Reduces Behavioral Response in Argentine Ants

**DOI:** 10.3390/insects11120871

**Published:** 2020-12-08

**Authors:** Jessica J. Maccaro, Brian A. Whyte, Neil D. Tsutsui

**Affiliations:** 1 Department of Entomology, University of California, Riverside, CA 92521, USA; 2 Department of Environmental Science, Policy, and Management, University of California, 130 Mulford Hall, #3114, Berkeley, CA 94720, USA; ba.whyte@berkeley.edu (B.A.W.); ntsutsui@berkeley.edu (N.D.T.)

**Keywords:** short-term, alarm pheromone, *Linepithema humile*, behavioral assay, Argentine ants

## Abstract

**Simple Summary:**

A significant challenge of chemical communication between ants is to maintain accurate communication of information in a variety of contexts. Argentine ants use volatile (airborne) compounds for a variety of functions, but one very important function is to elicit alarm via alarm pheromones. Given the importance of accurately responding to this signal, we expected Argentine ants to consistently show an alarm response to repeated exposure of alarm pheromones from their nestmates. However, we instead observed a reduction in their alarm behaviors over time. We speculate that a consistent response to repeated alarm signaling might require reinforcement from an actual alarming stimulus (e.g., the presence of predators or rival colonies). Argentine ants are considered a pest and several integrated pest management regimes use pheromones (i.e., mating disruption, aggregation pheromones, etc.) to reduce pest populations. Our results could be important to consider in the development of such control strategies because if ants habituate to their alarm pheromone over continuous exposure (without actually alarming stimuli) it might prove to be an ineffective strategy to repel them.

**Abstract:**

In this study we test whether Argentine ants (*Linepithema humile*) progressively reduce their response to a salient stimulus (alarm pheromone) with increased exposure over time. First, we used a two-chamber olfactometer to demonstrate three focal behaviors of Argentine ants that indicate an alarmed state in response to conspecific alarm pheromone and pure synthetic iridomyrmecin (a dominant component of *L. humile* alarm pheromone). We then measured how these behaviors changed after repeated exposure to conspecific alarm pheromone from live ants. In addition, we investigate whether there is a difference in the ants’ behavioral response after “short” (3 min) versus “long” (6 min) intervals between treatments. Our results show that Argentine ants do exhibit reduced responses to their own alarm pheromone, temporarily ceasing their response to it after four or five exposures, and this pattern holds whether exposure is repeated after “short” or “long” intervals. We suggest alarm pheromones may be perceived as false alarms unless threatening stimuli warrant a continued state of alarm. These results should be kept in mind while developing pheromone-based integrated pest management strategies.

## 1. Introduction

The societies of eusocial insects are typified by diverse and complex interactions among members of the colony. These interactions are facilitated by sophisticated systems of chemical communication that can be useful for myriad purposes, including recruitment [1], colony recognition [2], mate attraction and recognition [3], reproductive and caste recognition [4,5,6] task identification [7], repellency [8], brood care [9], or alarm [10,11].

A significant challenge of a heavily chemosensory lifestyle is the maintenance of olfactory acuity across a wide variety of contexts and circumstances. Desensitization to olfactory cues could play a role in olfactory acuity and be explained by a variety of mechanisms including sensory adaptation, habituation, or fatigue. Sensory adaptation is generally thought of as a peripheral nervous system process, whereas habituation is attributed to central nervous system processes [12]. Olfactory fatigue is considered a subcategory of severe sensory adaptation, where it becomes challenging and sometimes impossible to smell an odor for a given time [13]. These mechanisms can be difficult to disentangle from behavioral data alone, and this paper does not aim to do so. Rather, this paper will reveal a behavioral assay for testing insect response to olfactory cues over time, as well as demonstrate that Argentine ants lessen their response to alarm pheromone with increased intermittent exposure.

The Argentine ant *(Linepithema humile*) is a globally widespread and ecologically damaging invasive species [14]. As is true for ants generally, the coordination of their activities is primarily regulated by chemical signals and the study of their chemical communication has helped elucidate the mechanisms for their success (e.g., [2,10,15,16]). Since chemicals mediate insect behavior and coordination, there is interest in developing integrated pest control strategies that exploit these chemical signals to lower or repel pest populations (i.e., mating disruption [17,18] and mass trapping [19,20]). Our results might prove important to consider in the development of pheromone-based control strategies for Argentine ants.

Here, we describe the behavioral responses of Argentine ant workers to airborne alarm pheromones, test whether repeated exposure produces lessened olfactory acuity, and quantify behavioral responses under two different stimulation intervals. Since the alarm pheromone is an important signal of danger, one might expect that there would be strong selective pressure to ensure that this chemical is always perceived. Two volatile compounds (iridomyrmecin and dolichodial) that are found in the Argentine ant pygidial gland have been implicated as pheromones for alarm and defense [10]. We quantify three distinct behaviors that characterize the Argentine ant state of alarm by using synthetic iridomyrmecin [10] and live ant alarm pheromone. After verifying that these behavioral responses are qualitatively similar, we use live ants’ pheromones (as they are more biologically relevant) in short and long exposure interval tests to monitor ant response over time. Based on results from other systems, we hypothesize that these behavioral responses will dampen with repeated exposure to alarm pheromones, whether it be over short or long interstimulus intervals [21,22,23,24].

## 2. Materials and Methods

### 2.1. Ant Colony Collection

For all experiments, *Linepithema humile* were collected at Albany Bulb peninsula (Albany, CA, USA; 37.887329784–122.321498714) two days before each treatment. All ants were collected within a ten-meter radius and kept in a plastic bucket with substrate from the field. All collected ants were queenright. They were given 10% sucrose and distilled water on cotton balls once daily. All collections occurred between January and March of 2018.

### 2.2. Olfactometer

**Figure 1:**
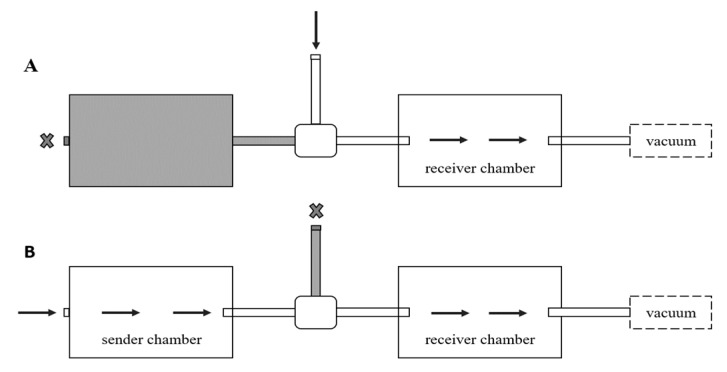
Olfactometer schematic. (**A**) Air flow during interstimulus points, when ambient air was being introduced into the receiver chamber. (**B**) Air flow during stimulus, when synthetic iridomyrmecin or alarm pheromone from living ants was being introduced into the receiver chamber.

To ensure that visual or mechanical cues did not influence the ants’ alarm behavior, we constructed a two-chamber olfactometer (Figure 1). The receiver chamber (15 × 9 mm; Sistema^®^ Plastic, Auckland, New Zealand) was connected to a vacuum valve with clear Poly Vinyl-Chloride (PVC) tubing (0.65 mm I.D. × 0.9 mm O.D. × 5.5 mm L) and the sender chamber (15 × 9 mm; Sistema^®^ Plastic) was connected to the receiver chamber via a 3-way forged brass ball valve with a lever handle (Nigo 180SS Series, Nigo Industrial Co., Ltd., Chang Hua City, Taiwan), with the 3rd opening of the valve open to the ambient air. This valve was held in place by a clamp so that when turned there would be no mechanical movement that would alarm the ants. For every experiment, an air flow meter was used to ensure the air flow rate in the sender chamber was always 2000 mL/min. The sender chamber was 380 cm^2^, so the equivalent of one chamber volume could be pulled to the receiver chamber in about 11.4 s. Since our measurement intervals were 30 s, this would guarantee that all air from the sender chamber would be present in the receiver chamber.

### 2.3. Defining Alarm Behaviors

In preliminary experiments, we observed the behavior of ants in response to several types of disturbance including mechanical (tapping vials that contained workers against a hard surface), exposure to other alarmed ants, or simply breathing on them. In all cases, we noted three specific behaviors that were associated with the alarm response, which correspond to previously determined ant alarm behaviors [10,25,26]. We quantified these behaviors in the subsequent experiments (below) to assess the alarm response of worker ants to stimuli. The first focal behavior was grooming (GROM), quantified as the number of grooming events within the designated measuring period. A grooming event was defined as any time an ant cleaned its antennae or legs, by placing them and/or drawing them through their mandibles. The second focal behavior was antennation frequency (AF), characterized by an ant touching any part of another ant with its antennae. The third focal behavior was antennal raising (AR), in which an ant would raise its antenna up into the air “in an exploratory way”, sniff their headspace, then lower them.

### 2.4. Testing Effect of Alarm Pheromone

#### 2.4.1. Single Exposure, Live Ant Pheromone

**Figure 2:**
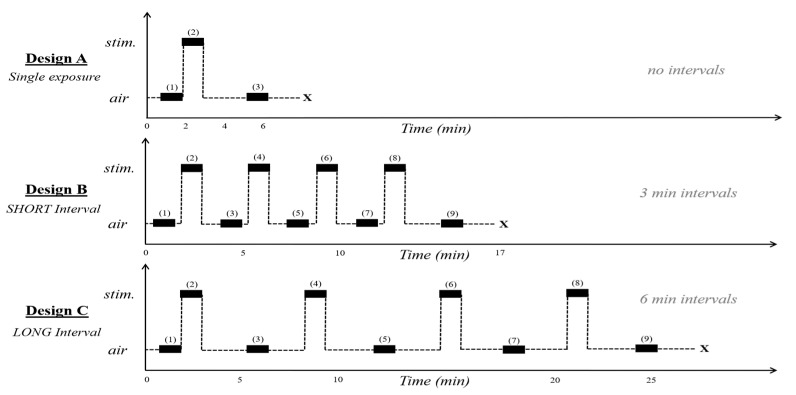
Designs for recording behavioral responses. Bolded boxes represent the 30 s time intervals when the three alarm behaviors were recorded, and the large “X” at the end of each line indicate when the experiment was ended. On the *y*-axis, “stim” represents when air passes through the sender chamber (Figure 1B), and “air” represents when ambient air is flowing directly into the receiver chamber (Figure 1A). (**A**) Single exposure to alarm pheromone from agitated ants or synthetic iridomyrmecin. (**B**) Multiple exposures to live ant alarm pheromone separated by “short” interstimulus intervals of 3 min. (**C**) Multiple exposures to live ant alarm pheromone separated by “long” interstimulus intervals of 6 min.

We first tested how ants responded to a single stimulation of alarm pheromone from alarmed nestmates. Twenty-five receiver ants were removed from the bucket with an aspirator and placed into a Petri dish with Fluon on the sides and a moist cotton ball to prevent dehydration. This was replicated for 20 separate Petri dishes (10 for the experimental treatment, and 10 for the control treatment). To begin the experiment, one Petri dish with 25 ants was placed in the receiver chamber, the lid was sealed, and airflow initiated (Figure 1). The video recording began after 5 min of acclimation in the receiver chamber. During this time, 100 stimulus ants were aspirated from the bucket and kept in the vial directly attached to the aspirator. Fifteen seconds before the stimulus interval, 100 stimulus ants were agitated by striking the vial against the counter ten times, then immediately placed into a Fluon-coated Petri dish inside the sender chamber. After closing the lid, the valve was switched to send airflow from the sender chamber to the receiver chamber for a 30 s stimulus interval (Figure 1B). The three alarm behaviors of the receiver chamber ants were recorded before the stimulus, during the stimulus, and 3 min after the stimulus (Figure 2A). Afterward, all ants from the receiver chamber and the sender chamber were discarded so that no ants would be re-used in further trials. These trials were interspersed with ten controls treatments performed in a random order, determined by a random number generator. The controls were performed in the same manner, but with an empty Fluon-coated Petri dish in the sender chamber instead of 100 agitated ants.

#### 2.4.2. Single Exposure, Synthetic Pheromone

We used the same method as above for this iridomyrmecin treatment, but the sender chamber contained synthetic iridomyrmecin instead of 100 irritated ants. Iridomyrmecin is one of the two components that comprise the argentine ant pygidial gland products, which have been shown to function as their alarm pheromone [10]. Synthetic iridomyrmecin in a solid state was received from the USDA ARS Invasive Insect Biocontrol and Behavior Laboratory (IIBBL). This solid isolate was dissolved in 95% ethanol at a concentration of 0.2 mg/mL, and 4 mL of this solution was pipetted into a glass vial (equal to 100 ants; following Choe et al. 2012 [16], although Welzel et al. 2018 [10] reports conflicting estimates). The ethanol was used as the solvent because it evaporates more quickly than the iridomyrmecin under nitrogen air flow, so that all that would be left in the vial was the iridomyrmecin. Once the ethanol evaporated, a thin waxy film of pure iridomyrmecin remained in the vial. The vial’s mouth rested on the valve in the sender chamber. The vial, being much larger, did not seal the valve; it was only placed this way to allow the air to be pulled out of the vial before pulling from other air in the sender chamber. For the control, an empty vial was filled with 4 mL of 95% ethanol and evaporated before placing it in the sender chamber in the same orientation. Data were collected in the same method as above (Figure 2A).

### 2.5. Repeated Exposures to Alarm Pheromone

To monitor the ants’ behavioral responses to alarm pheromone with increased exposure intervals we performed experiments in which 25 Argentine ant workers were stimulated four times for 30 s each (Figure 2). Two different interstimulus intervals (“short” and “long”) were chosen to test if the duration of time between stimuli changes the ants’ responses. This was replicated 10 times for long intervals as well as 10 times for short intervals. We hypothesized that longer interstimulus intervals would allow for greater recovery from odorant stimulation, and thus produce more responsive ants across an equivalent number of stimulation events.

#### 2.5.1. Short Intervals

We performed a “short interval” experiment in which Argentine ant workers were provided only a short rest period between exposures to alarm pheromone. For this experiment, the alarm stimulus was introduced at 3 min intervals, and behavioral data were recorded before, during, and after the stimulus (Figure 2B). During each interstimulus interval (Figure 2B, odd timepoints), 100 new ants were collected, alarmed, and then placed into the sender chamber at each stimulus point (Figure 2B, even timepoints). The ants in the receiver chamber remained the same throughout the experiment to monitor change their behavioral responses, across repeated stimulation events. The control was performed following the same design, but with an empty Fluon-coated Petri dish in the sender chamber.

#### 2.5.2. Long Intervals

We next performed a “long interval” experiment in which Argentine ants were exposed to the same number of alarm pheromone stimulations, but at 6 min intervals, twice as long as in the “short interval” experiment. Although these intervals were still fairly short, we wished to test whether the longer rest period provided enough time to recover following successive pheromone exposures, relative to behaviors observed in the short-interval experiments. As before, behavioral data were recorded before, during, and after the stimulus points (Figure 2C). Because the interstimulus intervals in this experiment were longer than in the short-interval habituation experiment, interstimulus behavioral data were recorded three minutes after each stimulus point (Figure 2C). The 100 new ants for each stimulus interval were collected during the last three minutes of the six-minute interval to ensure that the sender ants between both treatments were in the vial for the same amount of time. The control for this test was performed following the same procedure, but with an empty Fluon-coated Petri dish in the sender chamber.

### 2.6. Data Collection and Analysis

**Figure 3:**
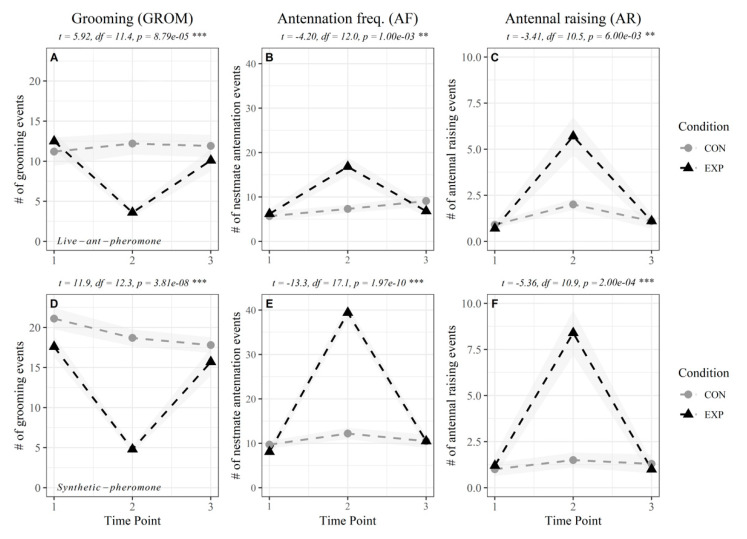
Response to single exposure. Behavioral responses to alarm pheromone emitted from agitated ants (**A**–**C**) or synthesized iridomyrmecin (**D**–**F**) in the sender chamber. Timepoint 2 (TP 2) is the stimulus point, when the experimental subjects received air from the sender chamber. Results from a Welsh two-sample t-test between the negative control (CON; ambient air) and stimulus (EXP; sender chamber air) at timepoint 2 are shown above each graph (** *p*-value ≤ 0.001, *** *p*-value ≤ 0.0001). Shaded ribbons around each line show standard error around the mean. For both the live ant pheromone and synthetic pheromone experiments, *n* = 10 replicate groups, each with 25 ants.

**Figure 4:**
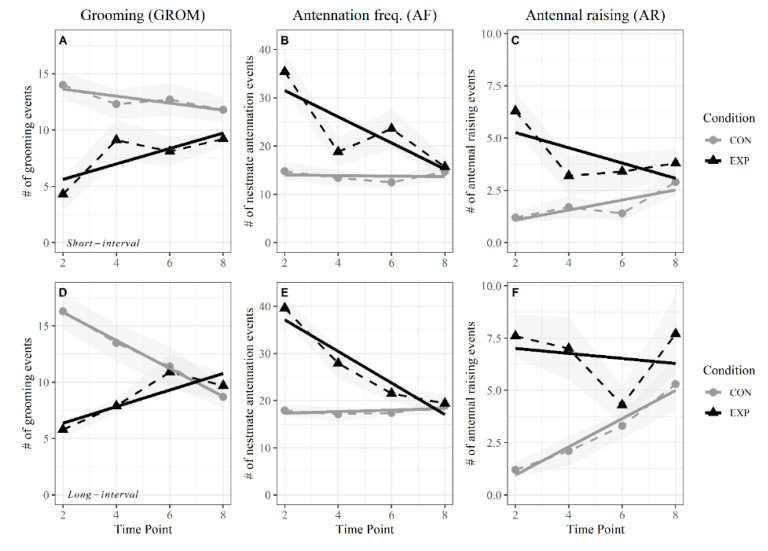
Patterns of reducing alarm response. Behavioral responses to repeated exposure of alarm pheromone over short (**A**–**C**) and long (**D**–**F**) intervals. Solid lines are linear models of the change in each behavior over the experimental duration. Dashed lines represent mean behavior surrounded by a shaded standard error ribbon. Only stimulus timepoints are included in these graphs. CON = ambient air, EXP = sender chamber air. For both the short- and long-interval experiments, *n* = 10 replicate groups, each with 25 ants.

The receiver chamber was filmed throughout the entire duration of each experiment using a Canon EOS 6D camera with an Electro-Focus (EF) 100 mm macro lens. Each video was later viewed in a VLC Media Player. Each of the three focal behaviors were quantified within 30 s intervals before, during, and after each stimulus point. Within each interval of measurement, the number of behavioral events were recorded, not the number of individuals performing the behavior. A summary of the data used in Figure 3 and Figure 4 can be found in the Supplemental Materials (Tables S1 and S2). The remaining figures (Figure 1 and Figure 2) were created in Microsoft PowerPoint 2016.

To test if stimulus events elicited significantly different behaviors in the single exposure experiments, we used a Welsh two-sample *t-*test (results reported in Figure 3). For the repeated exposure experiments, we used a repeated measures analysis of variance (ANOVA) testing the interaction of condition groups and timepoints and a between-trial effect (Model = Behavior ~ Condition × Timepoint + Error (Trial)) (Table 1). The between-trial effect tests was used if the condition groups showed significantly different behaviors between trials, while the within-trial effect tests was used if the condition groups showed significantly different behavioral changes over timepoints (Table 1).

## 3. Results

### 3.1. Single Exposure

When exposed to a single 30 s introduction of alarm pheromone from 100 sender ants, there was a significant increase in the number of receiver ants antennating each other (Figure 3C, black triangles) and raising their antennae (Figure 3D, black triangles) compared to their control groups at the same timepoint. At the same time, grooming appeared to be interrupted, as significantly fewer receiver ants performed this behavior after the stimulation (Figure 3B, black triangles). All behaviors at the final timepoint (3 min later) were not substantially different from the behaviors at the first timepoint (3 min before exposure), indicating a relaxation to a pre-disturbance behavioral state in this amount of time.

When receiver ants were exposed to the synthetic alarm pheromone component, iridomyrmecin, a single 30 s stimulus produced qualitatively similar behavioral responses to those seen after exposure to living agitated ants, but the magnitude of the behavioral changes were larger (Figure 3D–F). All behaviors were significantly different from those displayed in the ambient air control groups. The frequency of ants grooming decreased during stimulus (Figure 3F, black triangle), whereas the frequency of ants antennating each other (AF), and raising their antennae (AR) increased during stimulus (black triangle lines in Figure 3D–F, respectively). Similar to the live ant stimulus, the magnitude of all behaviors fell back to pre-stimulus levels 3 min after the stimulus.

While all ants used in these experiments were colony mates collected from the same ten-meter radius field site, there appear to be innate behavioral differences between these collected groups, even when sampled under the same circumstances. For instance, grooming behavior for control groups in Figure 3A,D were different, as well as antennation frequency in the single exposure (Figure 3B, timepoint 2) and repeated exposure groups (Figure 4B,E, timepoint 2). However, within experiment, the behavioral differences between control and experiment were consistent, supporting the same conclusions.

### 3.2. Short Intervals

In this experiment, the ants experienced four successive exposures to alarm pheromone, each interspersed by a 3 min rest period (ambient air flow, Figure 1). At the first stimulus, the behavioral response of ants to alarm pheromones from 100 sender ants was substantially different from their corresponding ambient air control groups (Figure 4A–C). However, the magnitude of the behavioral response difference between control and experiment groups decreased across successive exposures to alarm pheromone, approaching the levels seen in the ambient air control groups (Figure 4A–C). Specifically, AF and AR decreased across successive stimulation events (Figure 4B,C), whereas increased (Figure 4A). When compared using a Repeated Measures ANOVA, all behaviors showed significant interaction between condition groups over timepoints (Table 1), meaning the condition groups were significantly different in how their behaviors changed over time.

Grooming (GROM) started off less frequent in the experimental groups, but increased in frequency after repeated exposure, projected to occur at the same frequency as the control group after five stimulus points (Figure 4A, Table 2). The experiment only incorporated four stimulus points, but the linear models of experiment and control groups predicted the grooming behavior between the two groups would become similar if a fifth stimulus point was introduced. As for antennation (AF, AR) behaviors, these started off at high frequencies, decreasing to levels similar to their control groups in five or fewer stimulus points (Figure 4B,C, Table 2).

### 3.3. Long Intervals

The response of ants to stimulation in the experiment with longer intervals between stimuli (6 min) was similar overall to the results seen in the short-interval experiment. Specifically, the condition groups were significantly different in how their behaviors changed over time (Table 1), always starting at substantially different levels and then, after successive stimulations, approaching an estimated intercept around 4–5 stimulus points (Figure 4, Table 2). The frequency of grooming (GROM) behavior increased with repeated stimulus, whereas antennation (AF, AR) behaviors decreased (Figure 4). However, the sharp increase in antennal raising at the last stimulus point (Figure 4F) means we cannot be confident that the antennal raising behavior would continue to decrease over time as the linear model suggests.

Different from the short-interval results though, experiment and control linear models in the longer interval test usually intercepted after four stimulus points, with only antennal raising intercepting closer to five stimulus points (Table 2), likely due to the sharp increase in the last stimulus point (Figure 4F). Overall, the pattern of reducing behavioral responses to alarm stimulus over time is the same for both the short- and long-interval experiments.

## 4. Discussion

Outside of an experimental setting, a reduced response to alarm pheromone is likely influenced by many factors. There could be different mechanisms enabling a colony to remain aware of iridomyrmecin even after sustained exposure. Following the research of “response thresholds” in ants [27], there could be asymmetry in the sensitivity of ants collectively perceiving this chemical. Some ants may stop responding to alarm while others remain responsive. The ants could also only need a single induction of alarm response to alarm pheromone in order to appropriately react to alarm stimuli, and additional stimulation may not be useful, or could even be costly. Studies of the aphid *Myzus periscope* [23] have shown lower fitness after habituation to their alarm pheromone in the context of predators, but if predation was removed, habituation led to fitness increases. Lastly, it has been noted that *D. melanogaster* can dishabituate with mechanical stimulation [28] and their larva can dishabituate by being exposed to noxious fumes [22]. If habituation underlies the behavioral changes we observed in this experiment, Argentine ants may resist or reverse habituation to alarm when other stimuli of a threat to their nest are also present (i.e., exposure to light, rival colonies, predators, etc.). Further experimentation is needed to determine if our results demonstrate habituation, sensory deprivation, or fatigue.

When alarm pheromone is presented without an actual threat, as it was in the present study, the exposed workers may not continue responding to the signal (i.e., “the ant who cried wolf”), nor amplify it by producing additional alarm pheromone in response. Following this line of reasoning, it is not so surprising that ants respond less to such an important chemical signal, because if there is no context of danger to follow the signal, the pheromone is essentially a false alarm. Context or concentration could be necessary for alarm pheromone to mean “alarm” and not something else. However, we did not test how different concentrations of the alarm pheromone influenced alarm response, so we cannot deduce the interactions of quantity and context in determining the Argentine ant response to alarm pheromone. We can only suggest that context could prove to be necessary for this alarm pheromone to consistently induce an alarm and future research should aim to address this.

The results we report here show that Argentine ants decrease their response towards pheromones that are vital to their survival after repeated short-term exposure. In this study we solely demonstrate that the ants lessen their behavioral response to alarm pheromone over repeated exposure, but we do not directly address the mechanism which could be due to sensory adaptation, habituation, or fatigue. Regardless of alarm pheromone exposure, we can expect ants to show changing behavioral responses after being transported to a new environment (hence, the non-zero slopes we see in the control groups of Figure 4). However, these control groups show opposing, not similar, behavioral changes to the experimental groups, suggesting a different mechanism to explain these behavior trends over time. Future research would greatly complement this study by using electroantennography (EAG) to disentangle if ants reduce their alarm response due to habituation versus sensory adaptation to alarm pheromone. Additionally, future studies could consider how pairing an actual threat (e.g., intra- or interspecific competitors, predators, or parasitoids) with alarm pheromone affects behavioral responses.

The evolutionary success of social insects generally, and the invasive success of the Argentine ant in particular, stem in part from their chemical communication abilities. Consequently, our results could be important for the development of pheromone-based control methods in integrated pest management (IPM). Many pheromone-based baits are used for mating disruption or attract and kill techniques, using sex pheromone and aggregation pheromone, respectively [17,18,19,20]. However, research into ant pheromone baits, using trail pheromone to attract ants, has already been developed [29,30,31,32,33,34]. The trail pheromone has been shown effective when paired with an insecticide to attract and kill ants [29,32]. Trail pheromones are considered useful for short range communication and so alarm pheromone, as a method for long range communication, may overcome some of the current challenges to disrupting ants [11,35]. This has motivated researchers to develop alarm pheromone baits that can act at a distance to attract ants [36,37]. This may sound counter-intuitive that ants would be attracted to alarm signals, but alarm pheromones are often used by ants to initiate coordinated group defense behaviors [36]. Therefore, this appears to be a very promising avenue to manage ants in an agricultural setting [37]. However, if alarm pheromone (or any pheromone) is to be considered in the future to control ant populations, it will be important to take into account that continuous exposure might weaken the ant’s response and thus the efficacy of the bait. This weakened response has been shown in our study, as well as in several other insect species including other hymenopterans [21,22,23,24].

## 5. Conclusions

In this study, we hypothesized that Argentine ant alarm behaviors would remain consistent even after repeated exposure to their alarm pheromone. However, we found that Argentine ants reduce their response to alarm pheromone after repeated exposure in a matter of minutes. All three recorded behaviors (GROM, AF, AR) displayed by worker ants approached the control values after repeated exposure in both short-interval and long-interval tests. These results are qualitatively very similar to observations from *Drosophila melanogaster*. In response to ethanol vapor, for example, *D. melanogaster* displays a stereotypical olfactory “startle” response, but it is significantly reduced after four repeated pulses with interstimulus periods ranging from 3–18 min [21]. Similarly, the *D. melanogaster* olfactory jump response that occurs after 4 s exposure to benzaldehyde is reduced after 2–15 pulses separated by 0.25–20 min intervals [28].

Additionally, our results confirm that a behaviorally similar alarm response can be induced by either introducing air from a chamber of alarmed ants or using synthetic iridomyrmecin alone. These findings support the recent proposal that Argentine ant pygidial products (iridomyrmecin and dolichodial) function as the Argentine ant alarm pheromone [10]. In fact, the alarm response to synthetic iridomyrmecin appears even stronger than the live ant release of alarm pheromone (Figure 3). This could be due to the fact that the amount used in this experiment was equal to 100 ant-equivalents of iridomyrmecin [16], not necessarily the amount they release when alarmed. Therefore, we did not attempt to make quantitative comparisons between response to synthetic iridomyrmecin and live ant alarm pheromone, but rather show qualitatively that the behavioral response is similar. Personal observations using solid phase microextraction (SPME) as well as other reports [10] confirm that iridomyrmecin is certainly present in the headspace of alarmed ants and synthetic iridomyrmecin solutions. However, while we can indirectly estimate how much iridomyrmecin was available in the sender chamber during stimulus points, we did not record the quantity in the receiver chamber head space during each experiment.

## Figures and Tables

**Table 1:** Repeated Measures ANOVA on repeated exposure data. Condition refers to the treatment group (experiment versus control), while timepoint refers to the timepoints shown in Figure 4. Df = degrees of freedom, Sum.sq. = sum of squares, Mean.sq. = mean of squares, Pr|>F| = *p*-value. ANOVA Formula = (Behavior ~ Condition × Timepoint).

**Behavior (Short Intervals)**		**Df**		**Sum.sq**	**Mean.sq.**	**F. Value**	**Pr|>F|**
Grooming	Error: Trial	Condition	1	505.00	505.00	13.81	0.00158
		Residuals	18	658.40	36.60		
	Error: Within	Timepoint	3	29.60	9.88	0.89	0.45
		Condition: Timepoint	3	156.20	52.08	4.17	0.00541
		Residuals	54	596.90	11.05		
Antennation freq.	Error: Trial	Condition	1	1814.00	1814.50	14.82	0.00117
		Residuals	18	2203.00	122.40		
	Error: Within	Timepoint	3	1207.00	402.40	7.63	0.000244
		Condition: Timepoint	3	1074.00	358.00	6.78	0.000578
		Residuals	54	2850.00	52.80		
Antennal raising	Error: Trial	Condition	1	112.80	112.81	10.55	0.00446
		Residuals	18	192.40	10.69		
	Error: Within	Timepoint	3	26.94	8.98	3.03	0.03732
		Condition: Timepoint	3	52.54	17.51	5.90	0.00147
		Residuals	54	160.27	2.97		
**Behavior (Long Intervals)**		**Df**		**Sum.sq.**	**Mean.sq.**	**F. Value**	**Pr|>F| **
Grooming	Error: Trial	Condition	1	304.20	304.20	10.82	0.00408
		Residuals	18	506.30	28.13		
	Error: Within	Timepoint	3	49.10	16.35	1.15	0.337
		Condition: Timepoint	3	410.10	136.70	9.63	3.41 × 10^−6^
		Residuals	54	766.40	14.19		
Antennation freq.	Error: Trial	Condition	1	1720.50	1720.50	35.41	1.2 × 10^−5^
		Residuals	18	874.60	48.60		
	Error: Within	Timepoint	3	1192.00	397.20	12.81	1.96 × 10^−6^
		Condition: Timepoint	3	1302.00	434.10	14	7.21 × 10^−7^
		Residuals	54	1675.00	31.00		
Antennal raising	Error: Trial	Condition	1	270.10	270.11	9.44	0.00657
		Residuals	18	515.30	28.63		
	Error: Within	Timepoint	3	82.20	27.41	3.82	0.0149
		Condition: Timepoint	3	88.50	29.51	4.11	0.0107
		Residuals	54	388.00	7.19		

**Table 2:** Points at which alarmed ant behavior returns to resting ant behavior. “Time pt. int.” refers to the *x* axis value where Control and Experiment linear models (shown on Figure 4) intercept. “Stim pt. int” refers to the nearest stim point that the linear model intercepts round up to.

Behavior	Interval	Condition	Slope	Time pt. int.	Stim pt. int.
Grooming	Short	Control	−0.31	10.05	5
	Short	Experiment	0.68		
	Long	Control	−1.20	7.15	4
	Long	Experiment	0.73		
Antennation freq.	Short	Control	−0.06	8.58	5
	Short	Experiment	−2.71		
	Long	Control	0.16	7.63	4
	Long	Experiment	−3.35		
Antennal raising	Short	Control	0.24	9.92	5
	Short	Experiment	−0.36		
	Long	Control	0.67	9.68	5
	Long	Experiment	−0.12		

## Supplementary Materials

The following are available online at https://www.mdpi.com/2075-4450/11/12/871/s1, Table S1: Summary of stimulus point data used in Figure 3, Table S2: Summary of data used in Figure 4.
