# Supplementary material for: The Ant Who Cried Wolf? Short-Term Repeated Exposure to Alarm Pheromone Reduces Behavioral Response in Argentine Ants
Source: Insects. 2020 Dec 8;11(12):871. doi: 10.3390/insects11120871 (PMC7762586; doi:10.3390/insects11120871)
Supplement: Supplementary file 1 [file insects-11-00871-s001.pdf]

Supplementary Material

Table S1: Summary of stimulus point data used in Figure 3. Exposure type refers to whether the receiver ants were exposed to agitated ants ("Ants") or synthetic iridomyrmecin ("Irido"). Condition refers to control (CON) or treatment (EXP) group.

Exposure	Expo. Type	Condition	Stim. Point	Behavior	Mean	SD	Min	Max
Single	Ants	CON	2	Grooming	12.2	4.31535	4	18
Single	Ants	EXP	2	Grooming	3.6	1.57762	1	6
Single	Ants	CON	2	Antennation freq.	7.3	2.75076	3	11
Single	Ants	EXP	2	Antennation freq.	16.8	6.5963	6	29
Single	Ants	CON	2	Antennal raising	2	0.94281	1	3
Single	Ants	EXP	2	Antennal raising	5.7	3.30151	1	12
Single	Irido	CON	2	Grooming	18.7	3.36815	13	24
Single	Irido	EXP	2	Grooming	4.8	1.47573	3	7
Single	Irido	CON	2	Antennation freq.	12.2	4.02216	5	19
Single	Irido	EXP	2	Antennation freq.	39.4	5.08156	32	48
Single	Irido	CON	2	Antennal raising	1.5	1.2693	0	3
Single	Irido	EXP	2	Antennal raising	8.4	3.86437	3	15

Table S2: Summary of data used in Figure 4. Intervals refers to whether the ants received alarm stimuli at 3-min ("Short") or 6-min ("Long") intervals. Condition refers to control (CON) or treatment (EXP) group.

Exposure	Intervals	Condition	Time Point	Behavior	Mean	SD	Min	Max
Repeated	Short	CON	2	Grooming	14	3.82971	8	19
Repeated	Short	CON	4	Grooming	12.3	4.16467	8	21
Repeated	Short	CON	6	Grooming	12.7	4.62	6	21
Repeated	Short	CON	8	Grooming	11.8	3.52136	5	16
Repeated	Short	EXP	2	Grooming	4.3	4.3729	1	16
Repeated	Short	EXP	4	Grooming	9.1	5.70477	2	18
Repeated	Short	EXP	6	Grooming	8.1	4.28045	2	15
Repeated	Short	EXP	8	Grooming	9.2	1.93218	7	12
Repeated	Short	CON	2	Antennation freq.	14.8	5.88407	7	23
Repeated	Short	CON	4	Antennation freq.	13.4	5.27468	5	21
Repeated	Short	CON	6	Antennation freq.	12.5	3.71932	8	19
Repeated	Short	CON	8	Antennation freq.	14.7	5.90762	7	25
Repeated	Short	EXP	2	Antennation freq.	35.4	11.7303	21	63
Repeated	Short	EXP	4	Antennation freq.	18.8	9.30711	10	40
Repeated	Short	EXP	6	Antennation freq.	23.6	13.0996	7	43
Repeated	Short	EXP	8	Antennation freq.	15.7	7.3794	6	34
Repeated	Short	CON	2	Antennal raising	1.2	0.91894	0	3
Repeated	Short	CON	4	Antennal raising	1.7	1.70294	0	6

Repeated	Short	CON	6	Antennal raising	1.4	1.07497	0	3
Repeated	Short	CON	8	Antennal raising	2.9	2.079	0	7
Repeated	Short	EXP	2	Antennal raising	6.3	2.79086	1	11
Repeated	Short	EXP	4	Antennal raising	3.2	3.01109	0	10
Repeated	Short	EXP	6	Antennal raising	3.4	2.87518	0	9
Repeated	Short	EXP	8	Antennal raising	3.8	2.20101	0	6
Repeated	Long	CON	2	Grooming	16.3	4.96767	7	25
Repeated	Long	CON	4	Grooming	13.5	5.27573	7	24
Repeated	Long	CON	6	Grooming	11.4	5.64112	7	24
Repeated	Long	CON	8	Grooming	8.7	3.0203	4	12
Repeated	Long	EXP	2	Grooming	5.8	2.39444	4	12
Repeated	Long	EXP	4	Grooming	7.9	1.91195	6	11
Repeated	Long	EXP	6	Grooming	10.9	4.93176	5	19
Repeated	Long	EXP	8	Grooming	9.7	3.77271	3	17
Repeated	Long	CON	2	Antennation freq.	17.9	3.95671	9	23
Repeated	Long	CON	4	Antennation freq.	17.1	4.81779	8	25
Repeated	Long	CON	6	Antennation freq.	17.4	2.06559	15	22
Repeated	Long	CON	8	Antennation freq.	18.9	5.50656	13	29
Repeated	Long	EXP	2	Antennation freq.	39.6	6.58618	31	51
Repeated	Long	EXP	4	Antennation freq.	27.9	10.2572	11	45
Repeated	Long	EXP	6	Antennation freq.	21.5	5.56277	13	30
Repeated	Long	EXP	8	Antennation freq.	19.4	5.50151	9	29
Repeated	Long	CON	2	Antennal raising	1.2	1.13529	0	3
Repeated	Long	CON	4	Antennal raising	2.1	2.23358	0	8
Repeated	Long	CON	6	Antennal raising	3.3	1.94651	1	7
Repeated	Long	CON	8	Antennal raising	5.3	3.9172	2	12
Repeated	Long	EXP	2	Antennal raising	7.6	3.27278	3	14
Repeated	Long	EXP	4	Antennal raising	7	4.73756	3	17
Repeated	Long	EXP	6	Antennal raising	4.3	2.75076	0	7
Repeated	Long	EXP	8	Antennal raising	7.7	5.85093	0	16
